# Substrate-Assisted Visualization of Surfactant Micelles via Transmission Electron Microscopy

**DOI:** 10.3389/fchem.2019.00242

**Published:** 2019-04-11

**Authors:** Zekun Zhang, Kaitao Li, Rui Tian, Chao Lu

**Affiliations:** State Key Laboratory of Chemical Resource Engineering, Beijing University of Chemical Technology, Beijing, China

**Keywords:** transmission electron microscopy, visualization, surfactant micelles, layered double hydroxides, morphological evolution

## Abstract

The visualization of the micellar morphological evolution for surfactant has drawn much attention due to its self-assemble ability to fold into various structures. However, the direct observation of the soft materials with low atomic number has been hampered because of the poor scattering contrast and complex staining process by the traditional transmission electron microscopy (TEM) techniques. Herein, we reported a novel strategy to the visualization of surfactant micelles with the assistance of layered double hydroxides (LDHs) via TEM. Owing to the uniformly distributed metal ions and positive charges in the LDHs, the surfactant at the micelle-water interface reacted with LDHs to form a stabilized architecture through electrostatic and hydrogen-bond interactions. The morphologies of the surfactant can be clearly observed through the surfactant-LDHs architectures, exhibiting high contrast by TEM techniques. Significantly, the micellar evolutions involving the spherical, rodlike, and wormlike shapes were successfully distinguished. Our results may provide great possibilities and inspirations for the visualization for morphology of soft matters.

## Introduction

Surfactant is a widely applied soft material which exhibits self-assemble ability to fold into micelles with various structures (Jain and Bates, 2003; Stano and Luisi, 2010; Liu et al., 2018). The studies of the morphology and structure for micelles have drawn increasing attention due to their great significance in biological and material science (Landsmann et al., 2010; Wang et al., 2013; Hu and Chou, 2014). Transmission electron microscopy (TEM) is a valid technique widely applied to provide morphological aspects for visualization (Jung et al., 2003; O'Reilly et al., 2005; Honda et al., 2010). However, the observation for the surfactant micelles has been hampered due to the poor scattering contrast of the constituent elements with low atomic number (Egerton, 2013; Proetto et al., 2014). Although the supplemented attachments of TEM (such as liquid-cell and cryo-TEM) can provide supports for the morphological studies, this method suffered from sophisticated operation and sampling procedure (Geng and Discher, 2005; Parent et al., 2017; Zeng et al., 2017; Creatto et al., 2018; Stawski et al., 2018; Zhao et al., 2018; Suys et al., 2019). Therefore, it is a topic of significance to realize the visualization for the morphological evolution of the micelles during the formation process.

Staining is commonly done with heavy metal oxides (e.g., OsO_4_ and RuO_4_) for improving TEM contrast (Trent et al., 1983; Serizawa et al., 2000; Humphrey, 2009; Aramaki et al., 2017; Xu et al., 2017; Parker et al., 2018). Unsatisfactorily, the staining process usually comes along with inhomogeneity for staining segment, which might affect the true representation of the samples (Smith and Bryg, 2006). Moreover, the toxicity of the heavy metals has largely limited the application of staining. The fact has motivated us to explore an environmentally friendly alternative to enhance the scattering contrast and assist the visualization of micelles (Claypool et al., 1997; Aso et al., 2013).

Layered double hydroxides (LDHs) are a class of 2D inorganic metallic layered materials composed of edge-sharing metal-hydroxide octahedral structure (Abellan et al., 2012; Tian et al., 2018). The versatile di- and trivalent metal cations distributed uniformly in the host layer with the tunable electropositivity derived from the trivalent metal ions (Wang and O'Hare, 2012; Tian et al., 2015). The constituent metal elements, positively-charged layers and the abundant active sites of LDHs provided great possibilities for the construction of functional composites through electrostatic interaction and hydrogen bonds (Rogez et al., 2011; Fan et al., 2014). These superiorities inspired us to explore the potential of LDHs to assist the visualization of surfactant micelles by TEM technique.

**Figure 1:**
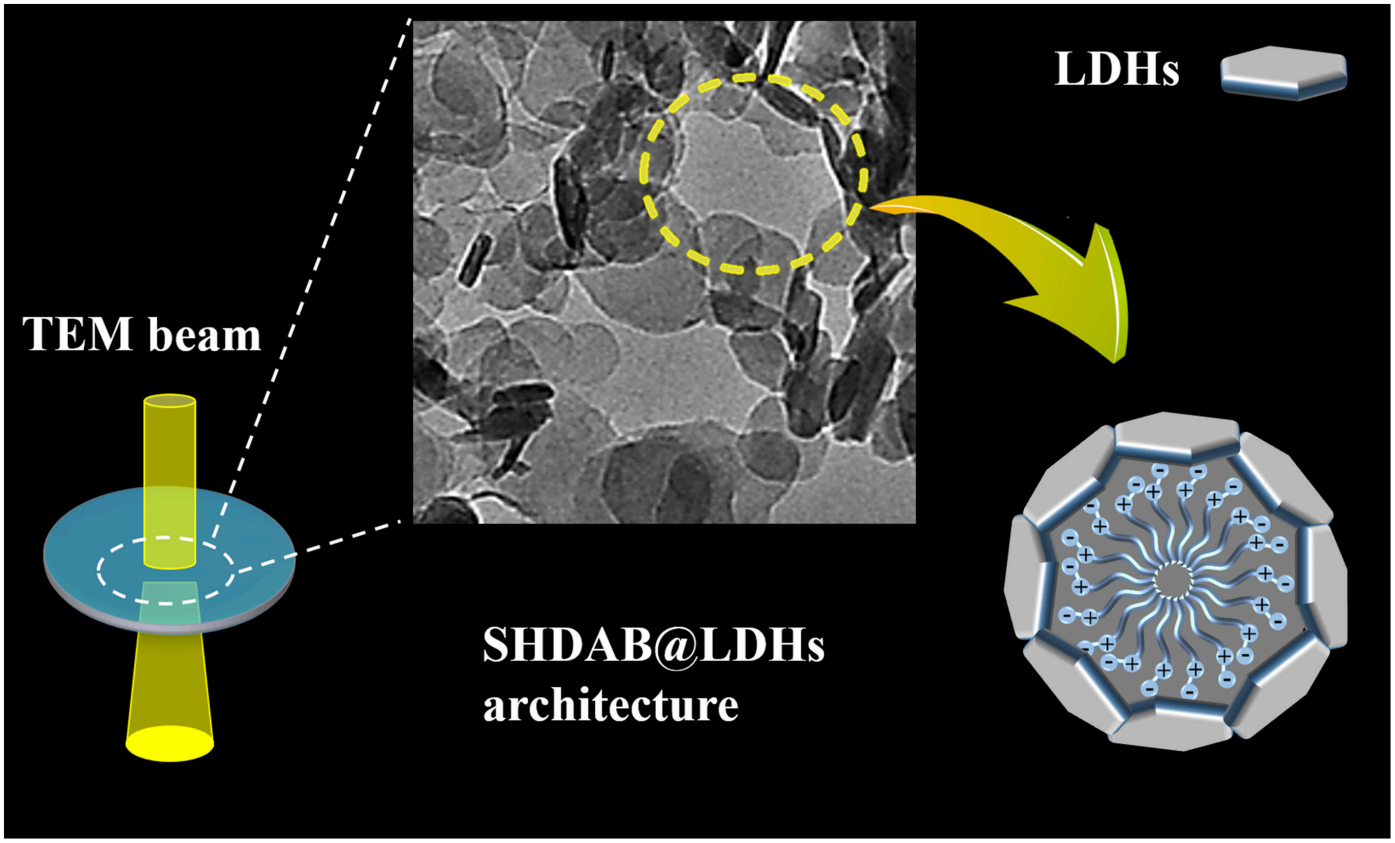
Schematic representation of visualization for surfactant micelles with the assist of LDHs via TEM imaging.

In this study, we demonstrated the visualization of surfactant micelles with the assist of LDHs via traditional TEM. An amphoteric surfactant *N*-hexadecyl-*N,N*-dimethyl-3-ammonio-1-propanesulfonate (SHDAB) with sulfonate anion in the head was employed. The functional groups at the micelle-water interface could react with LDHs to form a stabilized SHDAB@LDHs architecture by electrostatic and hydrogen-bond interactions (Figure 1). Owing to the capillary-compensated flow during droplet evaporation process, the LDHs nanoplates deposited at the edge of the droplet as “coffee-ring” (Deegan et al., 1997; Parneix et al., 2010; Cui et al., 2012) (Scheme S1). Visualization images with excellent contrast were achieved for SHDAB@LDHs architecture through TEM techniques. More importantly, the whole micellar morphological transition involving the spherical, rodlike and wormlike shapes could be effectively recognized with the continuously increased concentration of SHDAB (Scheme S2). The sizes of the micelles in the evolution identified with the data obtained from atomic force microscope (AFM). Therefore, it is anticipated that LDHs-assisted visualization of surfactant micelles may open new possibilities of TEM applications, especially for the soft materials.

## Methods

### Materials, Reagents, and Instruments

All reagents in experiments are of analytical grade and used without further purification. NaOH, Al(NO_3_)_3_·9H_2_O, Mg(NO_3_)_2_·6H_2_O are purchased from Beijing Chemical Reagent Company (Beijing, China). SHDAB is purchased from Tokyo Chemical Industry Co. Ltd (Tokyo, Japan). Ultra-pure water was obtained from the Milli-Q purification system (Barnstead, CA, USA).

Surface tension measurements of the SHDAB surfactant were measured on a Force Tensiometer (K100) by the Wilhelmy plate technique (Kruss, Germany). Electrical conductivity measurements were accomplished using an EC 215 conductivity meter (Shanghai Jingmi Instrumental Co., China). The ultrasonic treatment was implemented in an ultrasonic cleaning machine (Kunshan Ultrasonic Instrument Co. Ltd., China) with the frequency of 100%. TEM images were measured by the Tecnai G^2^20 (FEI Company, USA) with the accelerating voltage of 200 kV. The morphological studies of the LDHs were implemented on a scanning electron microscope (SEM Hitachi S-4700). The morphology data of SHDAB micelles were acquired on AFM by the NanoScope 9.1 (Bruker, Germany) instrument. X-ray diffraction (XRD) patterns of SHDAB, LDHs and SHDAB@LDHs architectures were measured on a D8 ADVANCE X-ray diffractometer (Bruker, Germany). FT-IR spectra were performed on a Nicolet 6700 (Thermo Electron). Zeta potentials of all the samples were recorded on a Malvern Zetasizer 3000HS nano-granularity analyzer. Isothermal titration calorimeter (ITC) was employed to study the interaction between SHDAB and LDHs on a Nano ITC (TA Instruments Waters, LLC, UT). 1.0 mL SHDAB aqueous (3.69 × 10^−4^ mol·L^−1^) was titrated into 0.25 mL LDHs (2.00 × 10^−3^ mol·L^−1^) in the measurement which was periodically calibrated with an internal electric heater. The heats of interaction during each injection were measured by integration of each titration peak using the ORIGIN software delivered with the ITC.

### Synthesis of MgAl-CO_3_-LDHs

According to the reported method (Gao et al., 2017; Tian et al., 2018), the LDHs were prepared with a few modifications. A mixed salt solution of Mg(NO_3_)_2_·6H_2_O (19.200 g, 0.075 mol) and Al(NO_3_)_3_·9H_2_O (9.375g, 0.025 mol) was dissolved in 150 mL water in a 250 mL flask. Subsequently, the prepared alkaline liquor of NaOH (8.000 g, 0.200 mol) was added to keep a constant pH value of 8.5. Then, the suspension was stirred for another 20 min, followed by transfer to a furnace tube at 110°C for 24 h. Finally, the precipitate was centrifuged, washed with water and stored at 4°C for further use.

### Preparation of Surfactant Micelles

The stock solution was prepared by dissolving SHDAB (0.017 g, 0.044 mol) in 30 mL ultra-pure water. Then, the SHDAB solution with different concentration (0.0295 mM, 0.059 mM, 0.295 mM, 1.48 mM) was prepared. After the ultrasonic treatment at 50°C for 4 h, the micelles with different morphologies were acquired.

### Synthesis of SHDAB@LDHs Architecture

Firstly, the LDHs suspensions were treated under ultrasonic to obtain a uniformly dispersed colloidal solution. Then, the as-prepared LDHs with different volumes (3.53, 7.06, 35.29, 176.45 μL) were added into the SHDAB surfactant micelles solution of different concentration (0.04 mM, 0.08 mM, 0.40 mM, 2.00 mM), respectively. Finally, the mixtures were treated under ultrasonic irradiation at 50°C for 4 h to prepare the SHDAB@LDHs architectures. In order to evaluate the morphological evolution of SHDAB micelles, *m*-SHDAB@LDHs architectures were prepared with the molecular ratio of SHDAB to LDHs determined as 0.295 to 0.400. The detailed compositions in *m*-SHDAB@LDHs architectures were listed in Table 1.

**Table 1:** The concentrations of SHDAB and LDHs in samples *m*-SHDAB@LDHs architectures.

**Samples**	**SHDAB/mM**	**LDHs/mM**
*m* = 0.0295	0.0295	0.04
*m* = 0.0590	0.0590	0.08
*m* = 0.295	0.295	0.40
*m* = 1.48	1.48	2.00

## Results and Discussion

### Characterizations of the Surfactant

**Figure 2:**
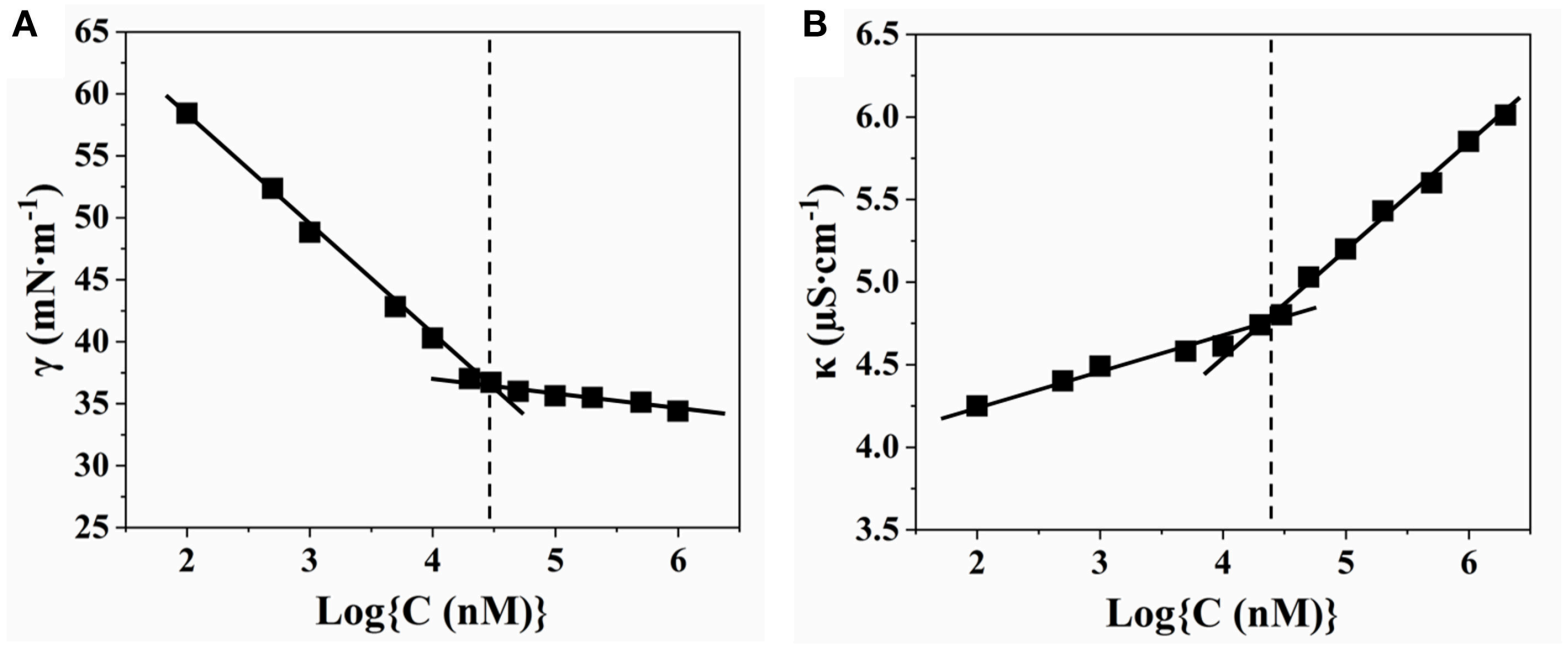
Relationship between the concentration of SHDAB with **(A)** surface tension (γ) and **(B)** conductivity (κ), respectively.

*N*-hexadecyl-*N,N*-dimethyl-3-ammonio-1-propanesulfonate (SHDAB) is a kind of betaine surfactant with negatively charged sulfonic acid in headgroup (Figure S1). To study the morphological transitions of SHDAB, the critical micelle concentration (CMC) has been investigated through the surface activities study (Claussen, 1967). The surface tension (γ) was plotted with the logarithm value of concentration for SHDAB. With the increased logarithm value of concentration for SHDAB, the surface tension of SHDAB decreased sharply at first and reached a relatively constant value after a marked change at 0.0295 mM for SHDAB (Figure 2A). The phenomenon was attributed to the presence of SHDAB monomers which preferred to adsorb at the water-air interface when the concentrations were below the CMC. When the adsorption at the water-air interface got saturated with the increased SHDAB monomers, the SHDAB tended to form micelles in the solution, exhibiting an almost constant surface tension. The inflection point from Figure 2A demonstrated that the CMC of SHDAB was 0.0295 mM. Moreover, the value of CMC was also investigated by conductivity measurements (Guégan et al., 2016). Figure 2B showed that the conductivity (κ) grew linearly with the logarithm value of concentration for SHDAB increased. Then, a slow growth was observed when the concentration of SHDAB was larger than 0.0295 mM. Such a turning point manifested the formation of micelles was determined as CMC for SHDAB, in agreement with that obtained by surface tension measurement. Notably, the CMC value of 0.0295 mM for SHDAB accorded with previous work (Liu et al., 2013).

### Visualization of Surfactant Assisted by LDHs

**Figure 3:**
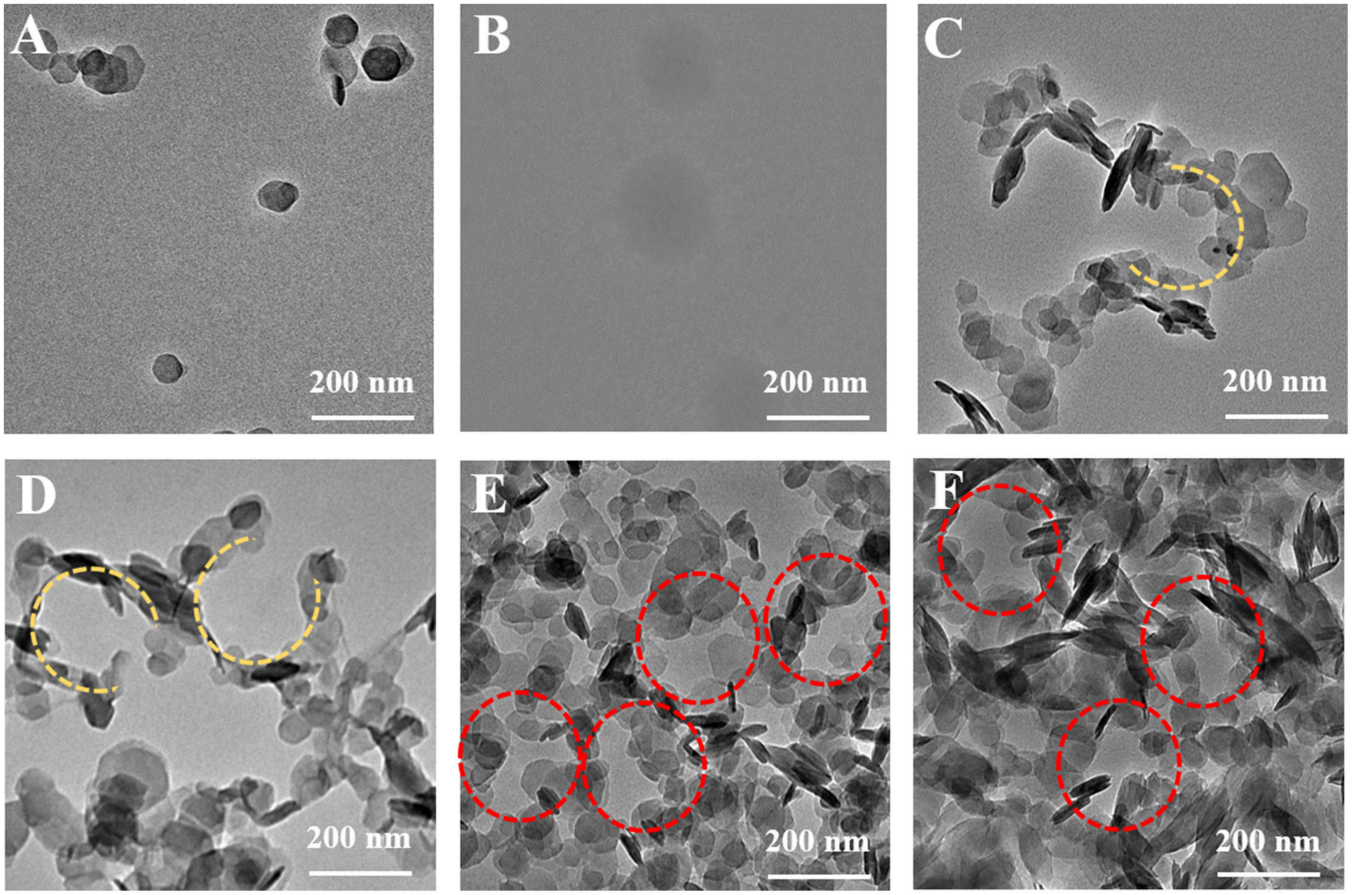
TEM images of **(A)** the LDHs, **(B)** the SHDAB micelles and **(C–F)** the SHDAB@LDHs architectures with increased concentration of LDHs in SHDAB aqueous solution (0.295 mM); from C to F: 0.04 mM, 0.08 mM, 0.40 mM and 2.00 mM, respectively.

To observe the morphological evolution of SHDAB micelles in different concentrations, MgAl-LDHs nanoplates were prepared via a hydrothermal method (Figure S2) (Gao et al., 2017; Tian et al., 2018). As shown in Figure 3A, the LDH particles were well-dispersed after ultrasonic irradiation. High contrast of inherent LDHs can be observed, as a result of the presence of metal elements (Mg and Al) in the host layers. On the contrary, TEM image of SHDAB micelles (0.295 mM) showed the blurred boundaries on account of weak scattering contrast by the constituent light elements (Figure 3B). In order to distinguish the SHDAB micelles, LDH particles were added into SHDAB to construct the SHDAB@LDHs architecture for visualization. Significantly, uniformly-distributed porous structure surrounded by continuous LDHs could be observed (Figure S3). The reason for the formation of the porous structure may be due to the fact that the positively charged LDHs were attached at the edges of the SHDAB micelles. During the evaporation process, the LDHs nanoparticles fell apart to the edge and accumulated at the border of the SHDAB@LDHs composites, and a “coffee-ring” was observed (Scheme S1) (Deegan et al., 1997; Parneix et al., 2010; Cui et al., 2012). The results demonstrated the success of LDH-assisted visualization of SHDAB by TEM technique.

The optimum conditions for the visualization of SHDAB micelles were carried out. Firstly, the LDHs and SHDAB micelles were mixed and treated under continuous stirring. However, the LDH particles aggregated randomly (Figure S3A), which failed to give a description for SHDAB micelles. With the assistance of heat treatment at 50°C to accelerate the interaction, porous structures were gradually formed (Figure S3B). The irregular morphology indicated the insufficient combination between LDHs and SHDAB micelles. It has been reported that the ultrasonic irradiation is a powerful strategy for the improvement of the reaction efficiency due to the active energy of ultrasound waves (Hasaninejed et al., 2013). Herein, the porous structure appeared under the condition of ultrasonic irradiation (Figure S3C). Moreover, heating treatment (50°C) was taken to optimize the conditions. The 0.295-SHDAB@LDHs architecture was successfully formed and stabilized with well-organized morphologies under ultrasonic irradiation along with thermal treatment (Figure S3D).

The quantities of LDHs in the visualization process were evaluated. LDHs with different concentrations (0.04 mM, 0.08 mM, 0.40 mM, 2.00 mM) were added into the SHDAB micelles (0.295 mM) under ultrasonic irradiation and thermal treatment. It can be observed that the LDHs nanoparticles accumulated around the SHDAB micelles gradually to form the SHDAB@LDH architecture (Figure 3). The porous structure was subsequently formed with the increased content of LDHs (Figures 3C,D), and a complete porous structures can be formed with the LDHs content of 0.40 mM (Figure 3E). Moreover, the excess increase of LDHs (up to 2.00 mM) led to the aggregated LDHs overlapped with the SHDAB@LDH architecture (Figure 3F). Therefore, to achieve the visualization of micelles, the molecular ratio of SHDAB to LDHs was determined as 0.295 to 0.400. In addition, the time of ultrasonic irradiation also exerted an influence on the SHDAB@LDHs architectures. With the prolonged time, LDHs got adjacent to electrical double layers formed at the micelle-water interface. As depicted in Figure S4, the 0.295-SHDAB@LDHs architecture exhibited excellent morphology until 4 h' treatment and achieved an equilibrium state afterwards. Therefore, a finely tuned visualization strategy for micelles has been successfully established, which showed high contrast and distinguished imaging effect.

### Visualization of the Morphological Evolution for the Surfactant

**Figure 4:**
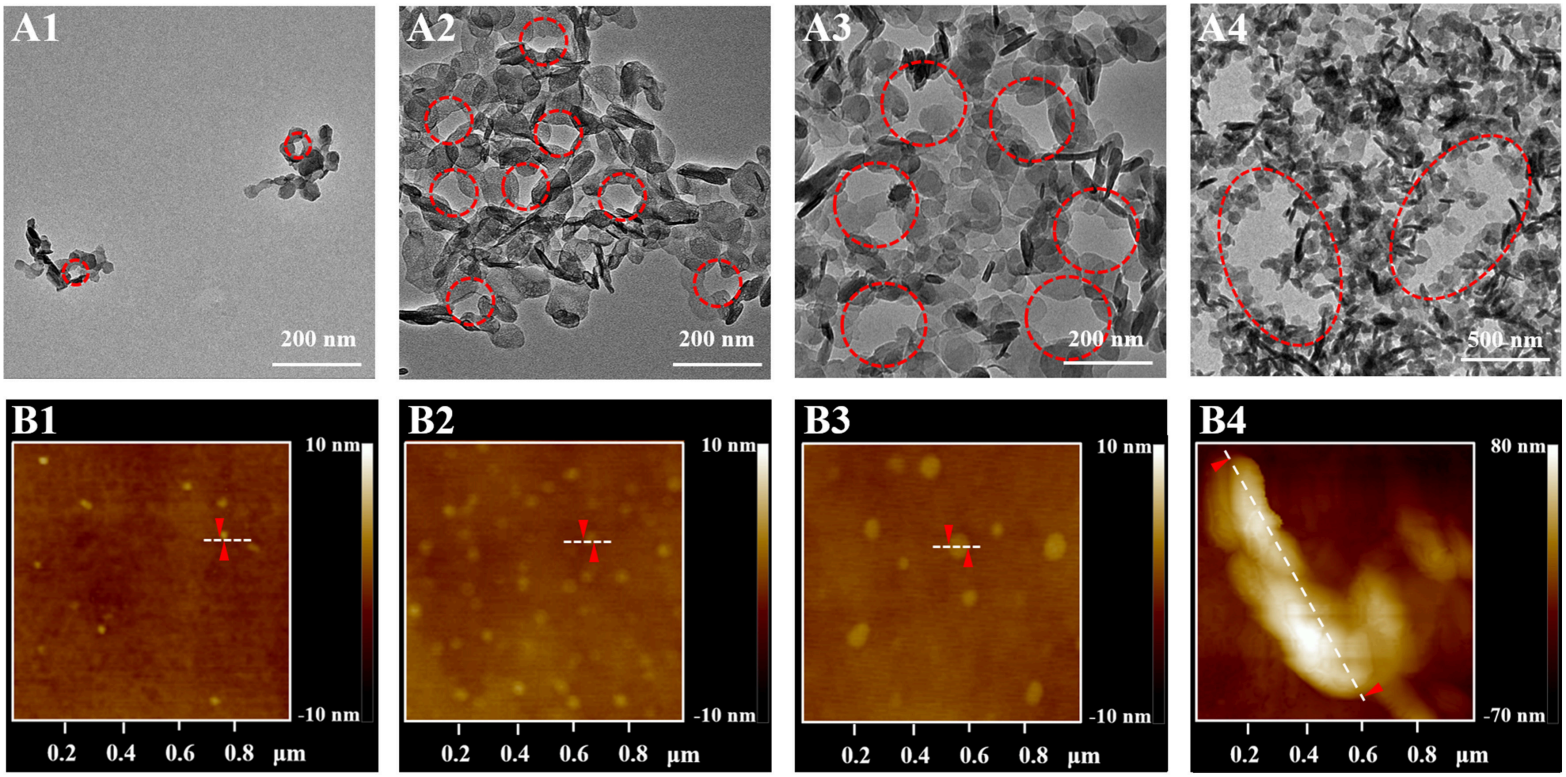
**(A)** TEM images of the proposed LDHs-assisted visualization of SHDAB, and **(B)** AFM investigations of SHDAB micelles; the concentration of SHDAB varied from 0.0295 mM (CMC), 0.0590 mM, 0.295 mM to 1.48 mM, respectively.

It has been reported that surfactants could be assembled into varied shapes when the concentrations of surfactant were larger than the CMC value (Shah et al., 2016). The SHDAB@LDHs architectures with increased concentration of SHDAB were prepared at the optimized ratio of SHDAB to LDHs (Table 1). With the concentration of SHDAB at the CMC value (0.0295 mM), a near-spherical porous structure was formed with an approximate radius of 26 nm, and the surfactant micelles were labeled with red dash line (Figure 4A1). The results indicated that the SHDAB micelles were surrounded by LDH nanoparticles. Interestingly, the porous architecture grew up to 58 nm with the concentration of SHDAB increased to 0.0590 mM (Figure 4A2). For the tenfold concentration of CMC value (0.295 mM), the SHDAB transformed from spherical to rodlike architecture with the size of 169 nm (Figure 4A3). Moreover, closely packed LDHs nanoplates around a lanky wormlike shape (length of 879 nm) can be observed with growing concentration of SHDAB to fiftyfold concentration of CMC (1.48 mM, Figure 4A4). It is noteworthy that the regulated activities of SHDAB from spherical, rodlike and wormlike structures can be visualized with the assist of LDHs by TEM imaging technique.

The dimensional and morphological properties of micelles in the transition process were testified by AFM. Figure 4B showed the AFM profile images of the pure SHDAB in the different concentrations from one to fifty times of CMC value. It was obvious that the micelles grew up from spherical to wormlike shape along with the increased concentrations. The section analysis was implemented to acquire the size of the micelles. The micelles changed from 30 nm, 62 nm and 178 nm of spherical shape to oval with the length of 903 nm when the concentration of LDHs changed from 0.0295 mM to 1.48 mM (Figure S5). The AFM data were in good conformity with the TEM images assisted by LDHs (Table S1). Moreover, the SHDAB@LDHs architectures can also be constructed by LDHs nanoparticles with smaller size (~25 nm, Figure S6), and the transformation of the micelles morphology can be visualized (Figure S7). These results demonstrated that the size of LDHs did not influence the visualization of SHDAB, and the proposed method showed good accuracy by the assistance of LDHs.

### Mechanism Studies

**Figure 5:**
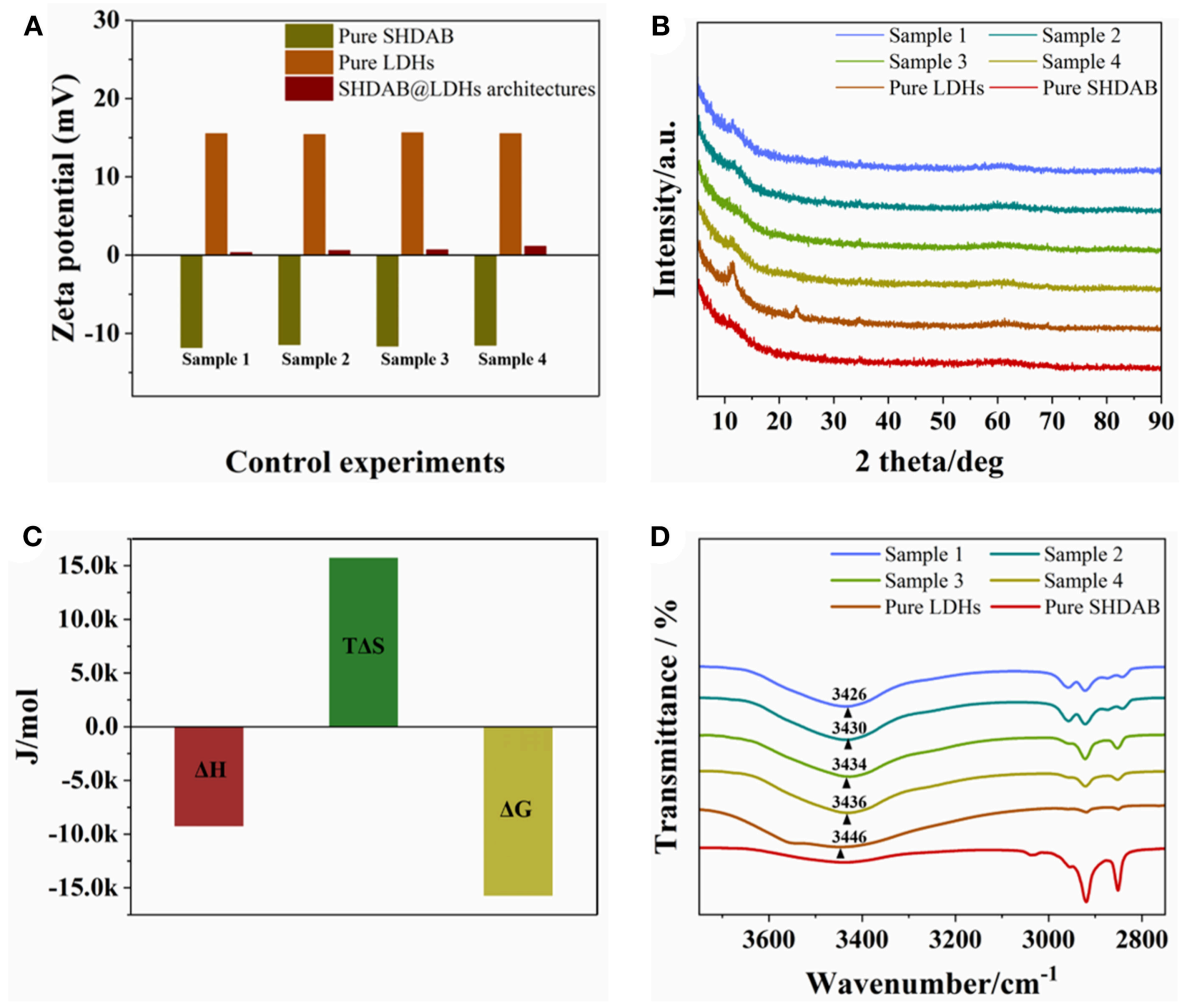
**(A)** Zeta potentials, **(B)** XRD patterns, **(C)** ITC, and **(D)** FT-IR measurements of SHDAB, LDHs and SHDAB@LDHs architectures. Sample 1 to 4 represented the 0.0295-SHDAB@LDHs, 0.0590-SHDAB@LDHs, 0.295-SHDAB@LDHs and 1.48-SHDAB@LDHs architectures, respectively.

In order to study the mechanism for the formation of SHDAB@LDHs architectures, several experiments were carried out. As illustrated from the structure of SHDAB in Figure S1, the SHDAB surfactant can self-assemble into micelles by the synergetic functions of the hydrophobic interaction between the alkane tail groups and electrostatic repulsion between the anionic head groups. As a result, the zeta potential value at the micelle-water interface of electrical double layer was −11.5 mV, indicating the stability of the SHDAB micelles with the negatively-charged groups exposed in solution (Figure 5A). Notably, the zeta potential of 15.5 mV for LDHs was beneficial for the electrostatic attraction toward the adjacent SHDAB micelles. The stabilized SHDAB@LDHs architecture with slight positive charge (0.3 mV) was obtained. The XRD measurements were implemented to study the structure of LDHs. The results demonstrated the unchanged interlayer space of LDHs, indicating that the SHDAB micelles were only attached at the surface of LDHs for the SHDAB@LDHs architectures (Figure 5B). To take a deep insight into the interaction between SHDAB micelles and LDHs, ITC measurement was carried out (Figure S8). The data indicated the simultaneous process for self-assembly of SHDAB micelles and the formation of the SHDAB@LDHs architectures (Kroflic et al., 2012). As shown in Figure 5C, the negative value of the free enthalpy showed the autonomous formation process of SHDAB@LDHs architectures (Zheng et al., 2016). Furthermore, the obvious vibration peaks in the range 3,650–3,200 cm^−1^ can be observed for the SHDAB@LDHs architectures. The peaks were blue-shifted in comparison with the FT-IR spectra for the pristine LDHs (Figure 5D). These results demonstrated the hydrogen bond interactions between quaternary ammonium cations of SHDAB and hydroxyl groups of LDHs. Therefore, both electrostatic and hydrogen bond interactions contributed to the formation of SHDAB@LDHs architectures for the visualization of SHDAB.

## Conclusion

In summary, we have presented an attractive approach for the visualization of surfactant SHDAB with the assist of LDHs via TEM imaging. Based on the electrostatic and hydrogen bond interactions between LDHs and SHDAB, well-organized SHDAB@LDHs architectures were formed. The morphologies of SHDAB surfactant can be distinguished with high scattering contrast and clear boundary, as a result of the environmentally friendly staining by LDHs. Notably, the morphological evolution involving spherical, rodlike and wormlike shapes can be witnessed, in accordance with AFM measurements. Therefore, our facile strategy opens up viable possibilities for the direct visualization of soft materials.

## Author Contributions

ZZ, RT, and CL conceived the experiments. ZZ and KL carried out the experiments. ZZ, RT, and CL contributed to data analysis and writing of this manuscript. All the authors have reviewed the manuscript and agreed to its publication.

### Conflict of Interest Statement

The authors declare that the research was conducted in the absence of any commercial or financial relationships that could be construed as a potential conflict of interest.

## References

[B1] Abellan G. Coronado E. Manti-Gastaldo C. Ribera A. Sanchez-Royo J. F. (2012). Layered double hydroxide (LDH)–organic hybrids as precursors for low-temperature chemical synthesis of carbon nanoforms. Chem. Sci. 3, 1481–1485. 10.1039/c2sc01064j

[B2] Aramaki K. Iwata C. Mata J. Maehara T. Aburano D. Sakanishi Y.. (2017). One-step formulation of nonionic surfactant bicelles (NSBs) by a double-tailed polyglycerol-type nonionic surfactant. Phys. Chem. Chem. Phys. 19, 23802–23808. 10.1039/c7cp02585h 28530285

[B3] Aso R. Kurata H. Namikoshi T. Hashimoto T. Kuo S. W. Chang F. C. (2013). Quantitative imaging of *T*_g_ in block copolymers by low-angle annular dark-field scanning transmission electron microscopy. Macromolecules 46, 8589–8595. 10.1021/ma4014934

[B4] Claussen W. F. (1967). Surface tension and surface structure of water. Science 156, 1226–1227. 10.1126/science.156.3779.1226 17792782

[B5] Claypool C. L. Faglioni F. Goddard W. A. Gray H. B. Lewis N. S. Marcus R. A. (1997). Source of image contrast in STM images of functionalized alkanes on graphite: a systematic functional group approach. J. Phys. Chem. B. 101, 5978–5995. 10.1021/jp9701799

[B6] Creatto E. J. Ceccacci F. Mancini G. Sabadini E. (2018). Effect of the hydrophobic tail of a chiral surfactant on the chirality of aggregates and on the formation of wormlike micelles. Langmuir 34, 13288–13295. 10.1021/acs.langmuir.8b02556 30350684

[B7] Cui L. Zhang J. Zhang X. Huang L. Wang Z. Li Y.. (2012). Suppression of the coffee ring effect by hydrosoluble polymer additives. ACS Appl. Mater. Interfaces 4, 2775–2780. 10.1021/am300423p 22545558

[B8] Deegan R. D. Bakajin O. Dupont T. F. Huber G. Nagel S. R. Witten T. A. (1997). Capillary flow as the cause of ring stains from dried liquid drops. Nature 389, 827–829. 10.1038/39827

[B9] Egerton R. F. (2013). Control of radiation damage in the TEM. Ultramicroscopy 127, 100–108. 10.1016/j.ultramic.2012.07.006 22910614

[B10] Fan G. L. Li F. Evans D. G. Duan X. (2014). Catalytic applications of layered double hydroxides: recent advances and perspectives. Chem. Soc. Rev. 43, 7040–7066. 10.1039/c4cs00160e 25001024

[B11] Gao R. Yan D. Evans D. G. Duan X. (2017). Layer-by-layer assembly of long-afterglow self-supporting thin films with dual-stimuli-responsive phosphorescence and antiforgery applications. Nano Res. 10, 3606–3617. 10.1007/s12274-017-1571-x

[B12] Geng Y. Discher D. E. (2005). Hydrolytic degradation of poly(ethylene oxide)-block-polycaprolactone worm micelles. J. Am. Chem. Soc. 127, 12780–12781. 10.1021/ja053902e 16159254 PMC2632957

[B13] Guégan R. Sueyoshi K. Anraku S. Yamamoto S. Miyamito N. (2016). Sandwich organization of non-ionic surfactant liquid crystalline phases as induced by large inorganic K4Nb6O17 nanosheets. Chem. Commun. 52, 1594–1597. 10.1039/c5cc08948d 26660331

[B14] Hasaninejed A. Kazerooni M. R. Zare A. (2013). Room-temperature, catalyst-free, one-pot pseudo-five-component synthesis of 4,4-(arylmethylene)bis(3-methyl-1-phenyl-1H-pyrazol-5-ol)s under ultrasonic irradiation. ACS Sustainable Chem. Eng. 1, 679–684. 10.1021/sc400081c

[B15] Honda S. Yamamoto T. Tezuka Y. (2010). Topology-directed control on thermal stability: micelles formed from linear and cyclized amphiphilic block copolymers. J. Am. Chem. Soc. 132, 10251–10253. 10.1021/ja104691j 20662503

[B16] Hu D. Chou K. C. (2014). Re-evaluating the surface tension analysis of polyelectrolyte-surfactant mixtures using phase-sensitive sum frequency generation spectroscopy. J. Am. Chem. Soc. 136, 15114–15117. 10.1021/ja5049175 25300026

[B17] Humphrey C. D. (2009). Negative stain transmission electron microscopy of viruses and virus-like particles. Microsc. Microanal. 15, 376–377. 10.1017/S1431927609094446

[B18] Jain S. Bates F. S. (2003). On the origins of morphological complexity in block copolymer surfactants. Science 300, 460–464. 10.1126/science.1082193 12702869

[B19] Jung H. M. Price K. E. McQuade D. T. (2003). Synthesis and characterization of cross-linked reverse micelles. J. Am. Chem. Soc. 125, 5351–5355. 10.1021/ja0271983 12720448

[B20] Kroflic A. Sarac B. Rogac M. B. (2012). Thermodynamic characterization of 3-[(3-cholamidopropyl)-dimethylammonium]-1-propanesulfonate (CHAPS) micellization using isothermal titration calorimetry: temperature, salt, and pH dependence. Langmuir 28, 10363–10371. 10.1021/la302133q 22686523

[B21] Landsmann S. Lizandara-Pueyo C. Polarz S. (2010). A new class of surfactants with multinuclear, inorganic head groups. J. Am. Chem. Soc. 132, 5315–5321. 10.1021/ja1011178 20302305

[B22] Liu F. Xiao J. W. Garamus V. M. Almásy L. Willumeit R. Mu B.. (2013). Interaction of the biosurfactant, surfactin with betaines in aqueous solution. Langmuir 29, 10648–10657. 10.1021/la400683u 23865739

[B23] Liu H. Wang R. Wei J. Cheng C. Zheng Y. Pan Y.. (2018). Conformation-directed micelle-to-vesicle transition of cholesterol-decorated polypeptide triggered by oxidation. J. Am. Chem. Soc. 140, 6604–6610. 10.1021/jacs.8b01873 29722260

[B24] O'Reilly R. K. Joralemon M. J. Wooley K. L. Hawker C. J. (2005). Functionalization of micelles and shell cross-linked nanoparticles using click chemistry. Chem. Mater. 17, 5976–5988. 10.1021/cm051047s

[B25] Parent L. R. Bakalis E. Ramírez-Hernández A. Kammeyer J. K. Park C. Pablo J.. (2017). Directly observing micelle fusion and growth in solution by liquid-cell transmission electron microscopy. J. Am. Chem. Soc. 139, 17140–17151. 10.1021/jacs.7b09060 29145727

[B26] Parker K. A. Li Y. Eshein A. Zhu S. Hampton C. M. Drummy L. F. (2018). Positive staining for improved contrast of macromolecules in transmission electron microscopy. Microsc. Microanal. 24, 1730–1731. 10.1017/S1431927618009133

[B27] Parneix C. Vandoolaeghe P. Nikolayev V. S. Quéré D. Li J. Cabane B. (2010). Dips and rims in dried colloidal films. Phys. Rev. Lett. 105, 266103. 10.1103/PhysRevLett.105.266103 21231686

[B28] Proetto M. T. Rush A. M. Chien M. P. Baeza P. A. Patterson J. P. Thompson M. P.. (2014). Dynamics of soft nanomaterials captured by transmission electron microscopy in liquid water. J. Am. Chem. Soc. 136, 1162–1165. 10.1021/ja408513m 24422495 PMC4021868

[B29] Rogez G. Massobrio C. Rabu P. Drillon M. (2011). Layered hydroxide hybrid nanostructures: a route to multifunctionality. Chem. Soc. Rev. 40, 1031–1058. 10.1039/c0cs00159g 21221445

[B30] Serizawa T. Takehara S. Akashi M. (2000). Transmission electron microscopic study of cross-sectional morphologies of core-corona polymeric nanospheres. Macromolecules 33, 1759–1764. 10.1021/ma9907486

[B31] Shah A. Shahzad S. Munir A. Nadagouda M. N. Khan G. S. Shams D. F.. (2016). Micelles as soil and water decontamination agents. Chem. Rev. 116, 6042–6074. 10.1021/acs.chemrev.6b00132 27136750

[B32] Smith R. W. Bryg V. (2006). Staining polymers for microscopical examination. Rubber Chem. Technol. 79, 520–540. 10.5254/1.3547949

[B33] Stano P. Luisi P. L. (2010). Achievements and open questions in the self-reproduction of vesicles and synthetic minimal cells. Chem. Commun. 46, 3639–3653. 10.1039/B913997D 20442914

[B34] Stawski T. M. Roncal-Herrero T. Fernandez-Martinez A. Matamoros-Veloza A. Kröger R. Benning L. G. (2018). “On demand” triggered crystallization of CaCO_3_ from solute precursor species stabilized by the water-in-oil microemulsion. Phys. Chem. Chem. Phys. 20, 13825–13835. 10.1039/C8CP00540K 29745416

[B35] Suys E. J. A. Warren D. B. Pham A. C. Nowell C. J. Clulow A. J. Benameur H.. (2019). A nonionic polyethylene oxide (PEO) surfactant model: experimental and molecular dynamics studies of Kolliphor EL. J. Pharm. Sci. 108, 193–204. 10.1016/j.xphs.2018.11.028 30502483

[B36] Tian R. Zhang S. T. Li M. W. Zhou Y. Q. Lu B. Yan D. P. (2015). Localization of Au nanoclusters on Layered Double Hydroxides nanosheets: confinement-induced emission enhancement and temperature-responsive luminescence. Adv. Funct. Mater. 25, 5006–5015. 10.1002/adfm.201501433

[B37] Tian R. Zhong J. P. Lu C. Duan X. (2018). Hydroxyl-triggered fluorescence for location of inorganic materials in polymer-matrix composites. Chem. Sci. 9, 218–222. 10.1039/c7sc03897f 29629090 PMC5869289

[B38] Trent J. S. Scheinbeim J. I. Couchman P. R. (1983). Ruthenium tetraoxide staining of polymers for electron microscopy. Macromolecules 16, 589–598. 10.1021/ma00238a021

[B39] Wang H. Y. Zhang L. M. Wang J. J. Li Z. Y. Zhang S. J. (2013). The first evidence for unilamellar vesicle formation of ionic liquids in aqueous solutions. Chem. Commun. 49, 5222–5224. 10.1039/c3cc41908h 23628851

[B40] Wang Q. O'Hare D. (2012). Recent advances in the synthesis and application of Layered Double Hydroxide (LDH) nanosheets. Chem. Rev. 112, 4124–4155. 10.1021/cr200434v 22452296

[B41] Xu H. Du N. Song Y. Song S. Hou W. (2017). Vesicles of 2-ketooctanoic acid in water. Soft Matter 13, 2246–2252. 10.1039/c6sm02665f 28255587

[B42] Zeng Z. Y. Zheng W. J. Zheng H. M. (2017). Visualization of colloidal nanocrystal formation and electrode-electrolyte interfaces in liquids using TEM. Acc. Chem. Res. 50, 1808–1817. 10.1021/acs.accounts.7b00161 28782932

[B43] Zhao M. Gao Z. Dai C. Zhang Y. Sun X. Gao M.. (2018). Investigation of active-inactive material interdigitated aggregates formed by wormlike micelles and cellulose nanofiber. J. Phys. Chem. B. 122, 10371–10376. 10.1021/acs.jpcb.8b06440 30170497

[B44] Zheng Y. You S. S. Ji C. D. Yin M. Z. Yang W. T. Shen J. (2016). Development of an amino acid-functionalized fluorescent nanocarrier to deliver a toxin to kill insect pests. Adv Mater. 28, 1375–1380. 10.1002/adma.201504993 26640174

