# Supplementary material for: Substrate-Assisted Visualization of Surfactant Micelles via Transmission Electron Microscopy
Source: Front Chem. 2019 Apr 11;7:242. doi: 10.3389/fchem.2019.00242 (PMC6470246; doi:10.3389/fchem.2019.00242)
Supplement: Supplementary file 1 [file Data_Sheet_1.doc]

Supporting Information for:

Substrate-assisted visualization of surfactant micelles *via* transmission electron microscopy

Zekun Zhang, Kaitao Li, Rui Tian* and Chao Lu*

*State Key Laboratory of Chemical Resource Engineering, Beijing University of Chemical Technology, Beijing 100029, China*


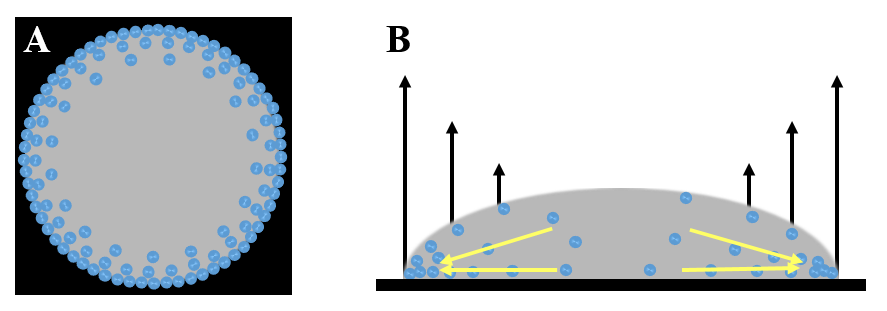


**Scheme S1.** Schematic representation of the "coffee-ring" after the evaporation processes from the (A) top-view and (B) side-view.


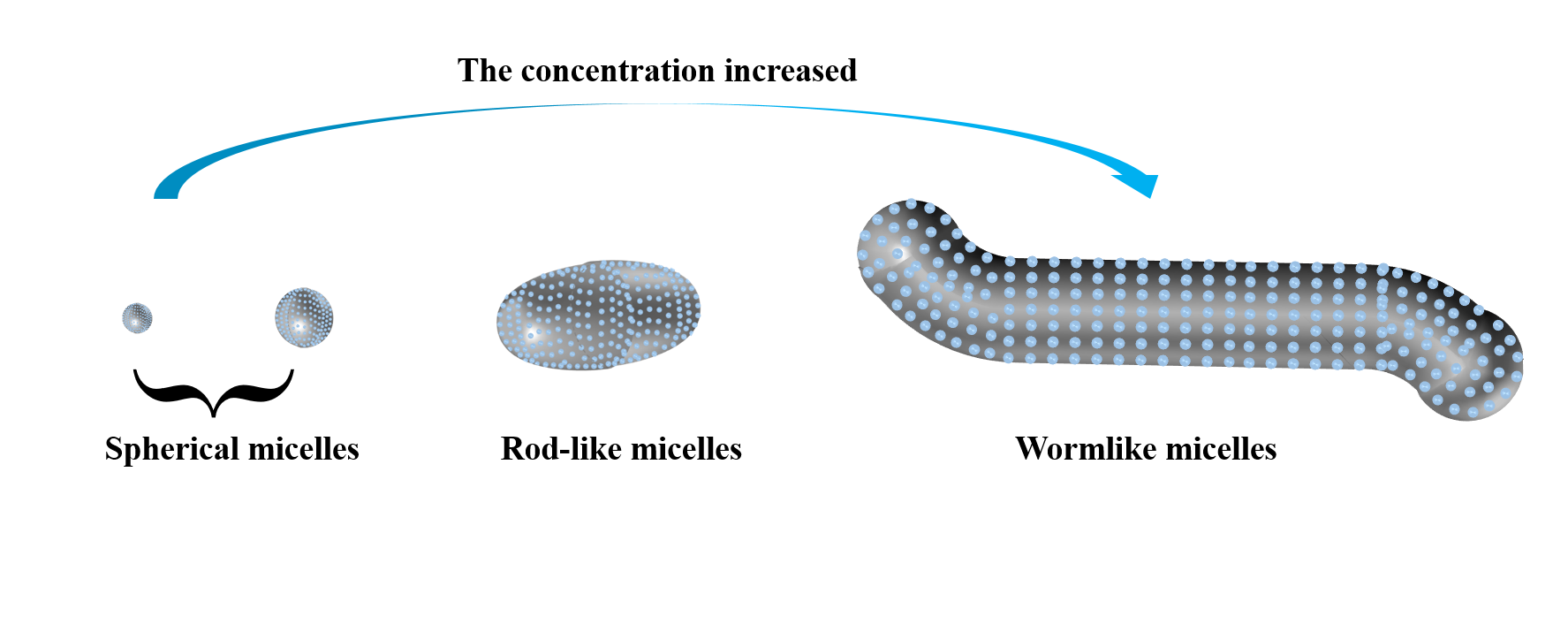


**Scheme S2.** Schematic diagrams for the micelle transitions from spherical, rodlike to wormlike shapes with the increased concentration of *N*-hexadecyl-*N,N*-dimethyl-3-ammonio-1- propanesulfonate (SHDAB).


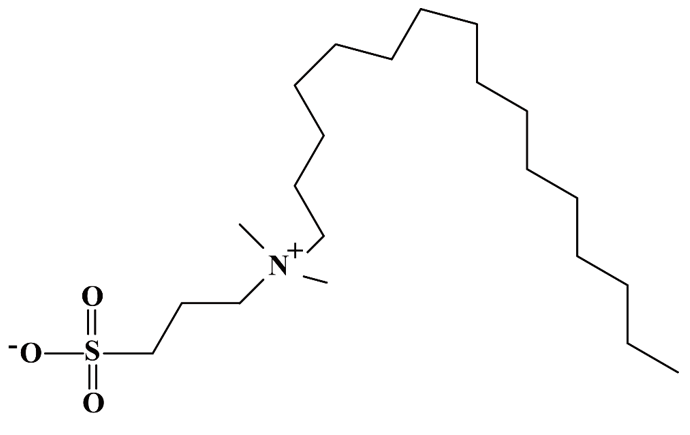


**Figure S1.** The structural formula of SHDAB.


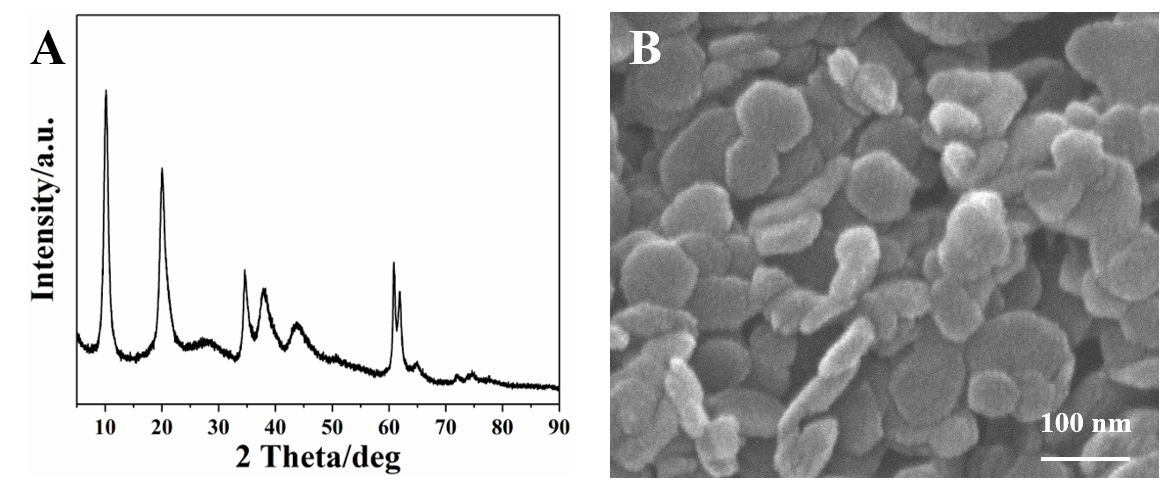


**Figure S2.** (A) X-ray diffraction (XRD) pattern and (B) scanning electron microscope image of layered double hydroxides (LDHs).


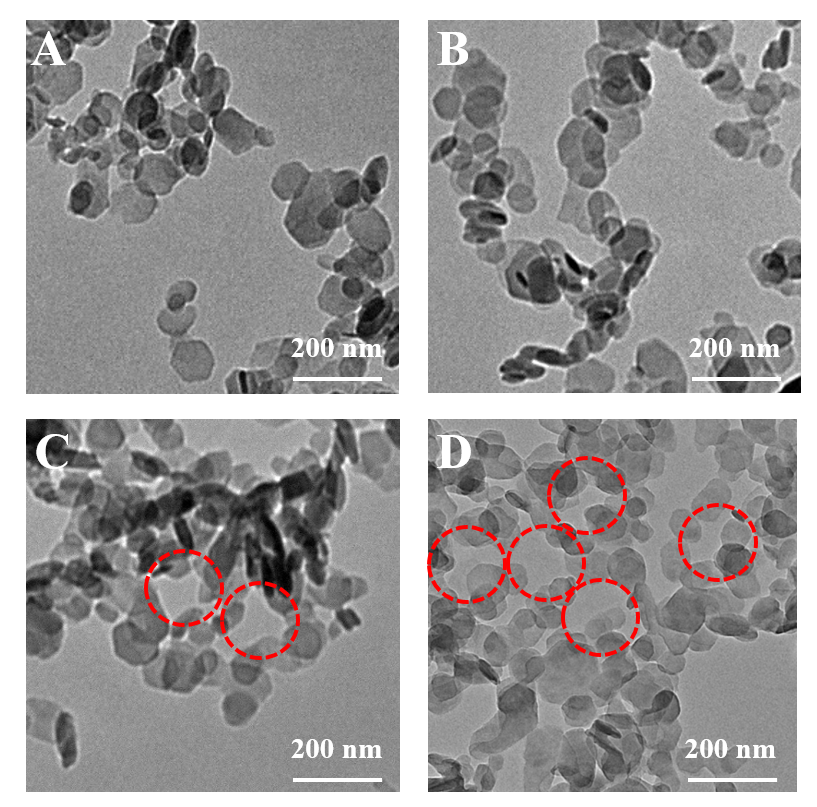


**Figure S3.** Transmission electron microscopy (TEM) images of the 0.295-SHDAB@LDHs composites under different treatments for (A) stirring, (B) stirring with heating at 50 ^o^C, (C) ultrasonic irradiation and (D) ultrasonic irradiation with heating at 50 ^o^C.


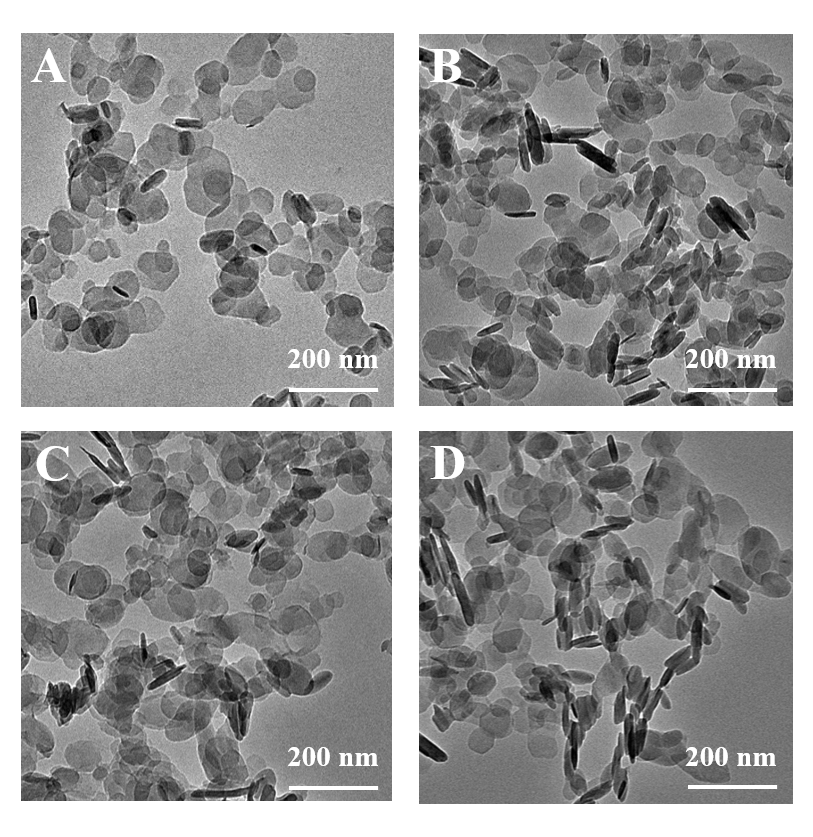


**Figure S4.** TEM images of the 0.295-SHDAB@LDHs architectures under different time for ultrasonic treatment; from A to D: 1 h, 4 h, 8 h and 12 h, respectively.


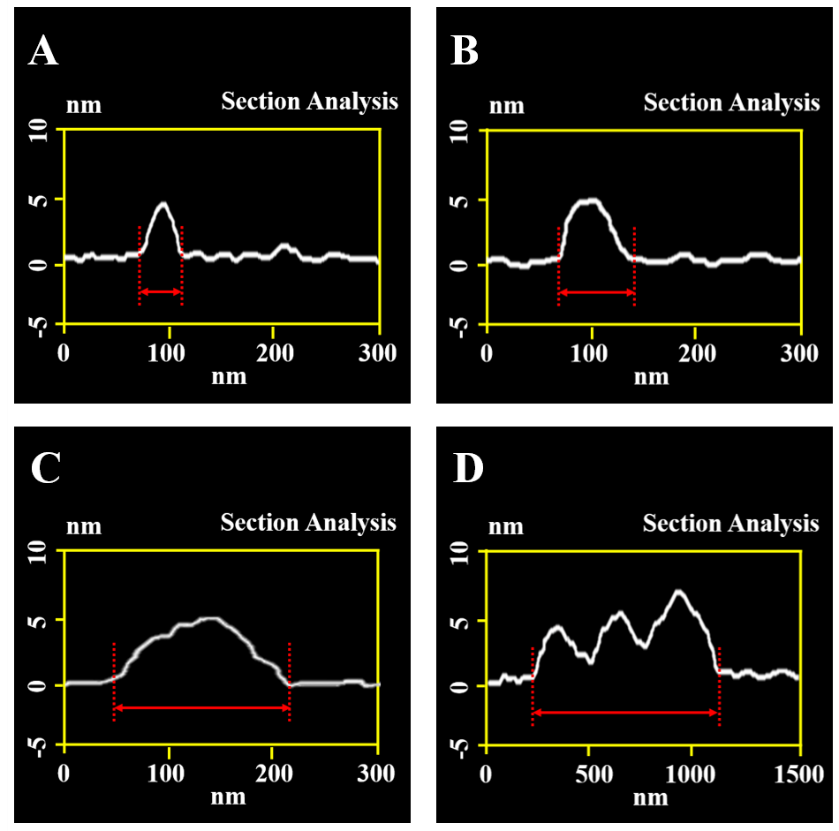


**Figure S5.** Section analysis in atomic force microscope measurements for SHDAB micelles; from A to D: the concentration of SHDAB varied from 0.0295 mM, 0.0590 mM, 0.295 mM to 1.48 mM, respectively.


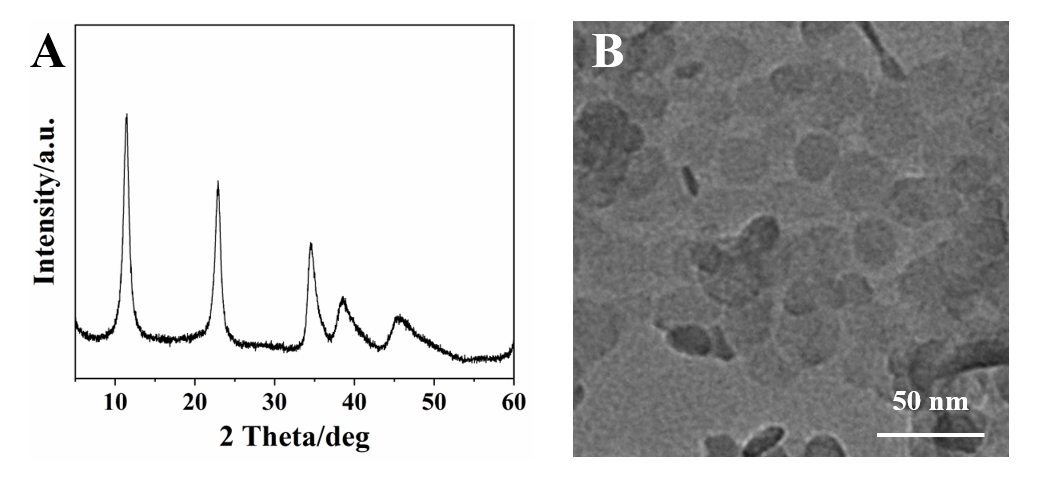


**Figure S6.** (A) XRD pattern and (B) TEM image of LDHs nanoparticles (the size is around 25 nm).


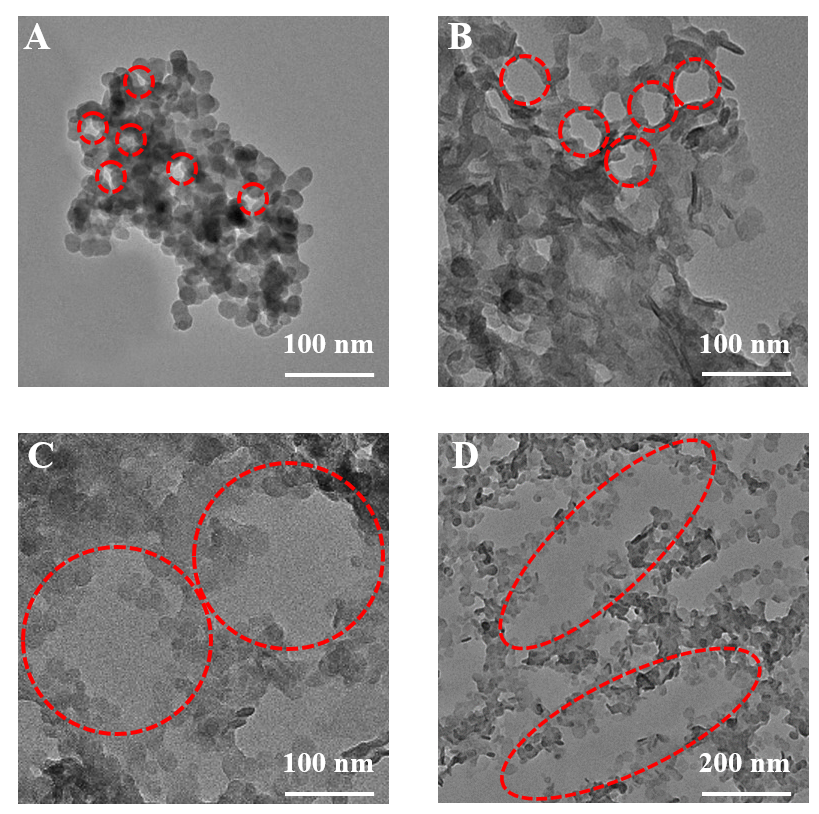


**Figure S7.** TEM images of the proposed LDHs-assisted visualization of SHDAB, the size of LDHs nanoparticles is about 25 nm, and the concentration of SHDAB varied from 0.0295 mM (CMC), 0.0590 mM, 0.295 mM to 1.48 mM, respectively.

**Table S1.** The sizes of the SHDAB micelles obtained from the TEM (*d*_1_) images of *m*-SHDAB@LDHs architectures and atomic force microscope (*d*_2_) images; the concentration of SHDAB varied from 0.0295 mM, 0.0590 mM, 0.295 mM to 1.48 mM, respectively.

| ***c* (SHDAB) /mM** | ***d*_1_/nm** | ***d*_2_/nm** |
| --- | --- | --- |
| 0.0295 | 26 | 30 |
| 0.0590 | 58 | 62 |
| 0.295 | 169 | 178 |
| 1.48 | 879 | 903 |


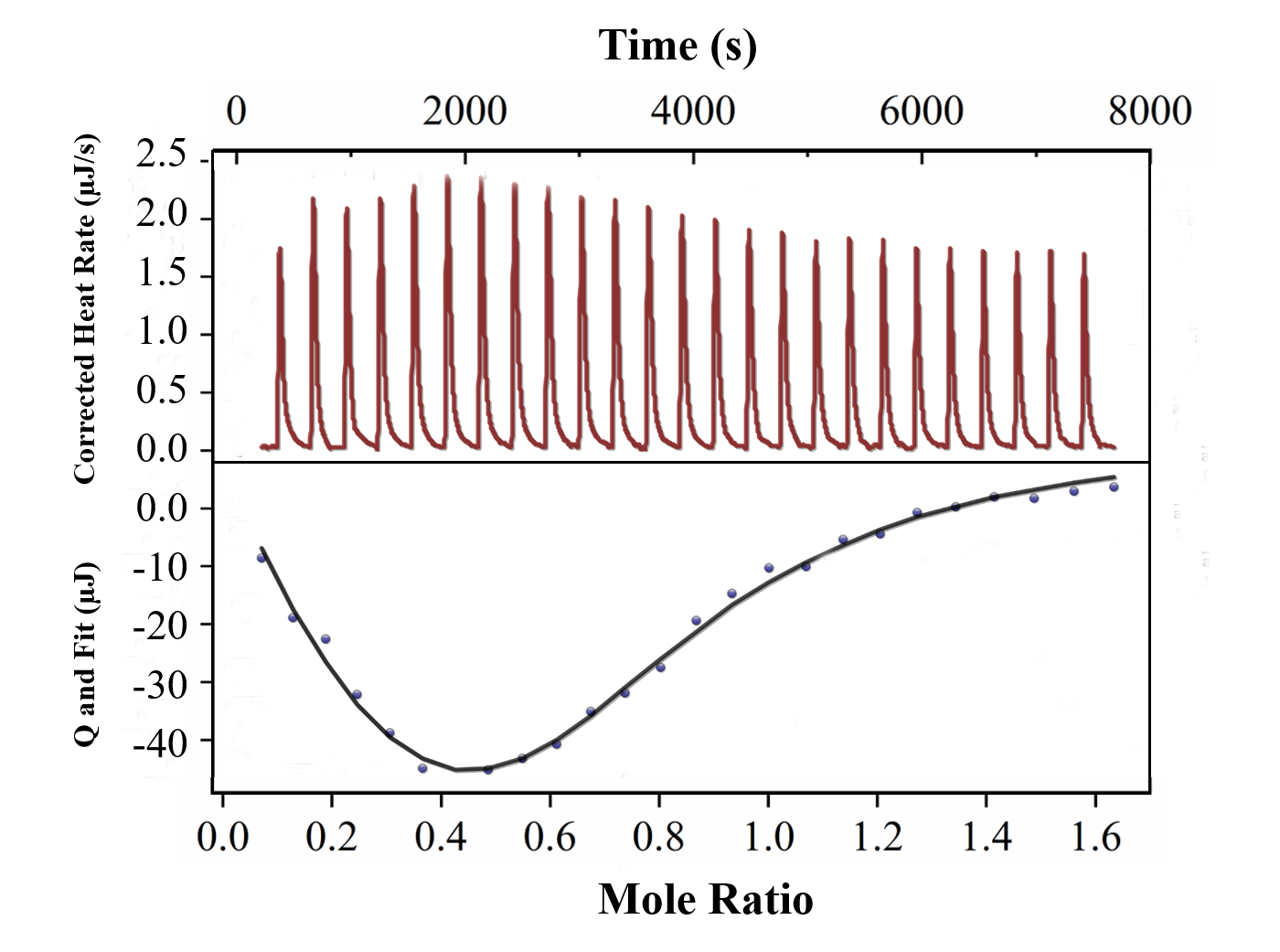


**Figure S8.** Isothermal titration calorimeter measurement for the interaction between SHDAB and LDHs.
